# Transcriptome analysis reveals pathogenesis-related gene 1 pathway against salicylic acid treatment in grapevine (*Vitis vinifera* L)

**DOI:** 10.3389/fgene.2022.1033288

**Published:** 2022-10-20

**Authors:** Faiz Ur Rahman, Irshad Ahmad Khan, Ali Aslam, Ruitao Liu, Lei Sun, Yandi Wu, Muhammad Muzammal Aslam, Asad Ullah Khan, Peng Li, Jianfu Jiang, Xiucai Fan, Chonghuai Liu, Ying Zhang

**Affiliations:** ^1^ Zhengzhou Fruit Research Institute, Chinese Academy of Agricultural Sciences, Zhengzhou, China; ^2^ Institute of Horticultural Sciences, University of Agriculture Faisalabad, Faisalabad, Pakistan; ^3^ Faculty of Agriculture and Veterinary Sciences, Superior University, Lahore, Pakistan; ^4^ The Key Laboratory for Crop Germplasm Resource of Zhejiang Province, Hangzhou, China

**Keywords:** Salicylic acid, PR1, white rot, Cis-elements, transcriptomics, grapevine

## Abstract

Salicylic acid (SA) is a well-studied phenolic plant hormone that plays an important role in plant defense against the hemi-biothrophic and biothrophic pathogens and depends on the living cells of host for the successful infection. In this study, a pathogenesis test was performed between *Vitis davidii* and *V. vinifera* cultivars against grape white rot disease (*Coniella diplodiella*). *V. davidii* was found to be resistant against this disease. SA contents were found to be higher in the resistant grape cultivar after different time points. RNA-seq analysis was conducted on susceptible grapevine cultivars after 12, 24, and 48 h of SA application with the hypothesis that SA may induce defense genes in susceptible cultivars. A total of 511 differentially expressed genes (DEGs) were identified from the RNA-seq data, including some important genes, *VvWRKY1/2, VvNPR1*, *VvTGA2*, and *VvPR1,* for the SA defense pathway. DEGs related to phytohormone signal transduction and flavonoid biosynthetic pathways were also upregulated. The quantitative real-time PCR (qRT-PCR) results of the significantly expressed transcripts were found to be consistent with the transcriptome data, with a high correlation between the two analyses. The pathogenesis-related gene 1 (*VvPR1*), which is an important marker gene for plant defense, was selected for further promoter analysis. The promoter sequence showed that it contains some important cis-elements (W-box, LS7, as-1, and TCA-element) to recruit the transcription factors *VvWRKY, VvNPR1*, and *VvTGA2* to express the *VvPR1* gene in response to SA treatment. Furthermore, the *VvPR1* promoter was serially deleted into different fragments (−1,837, −1,443, −1,119, −864, −558, −436, and −192 ) bp and constructed vectors with the GUS reporter gene. Deletion analysis revealed that the *VvPR1* promoter between −1837 bp to −558 bp induced significant GUS expression with respect to the control. On the basis of these results, the −558 bp region was assumed to be an important part of the *VvPR1* promoter, and this region contained the important cis-elements related to SA, such as TCA-element (−1,472 bp), LS7 (−1,428 bp), and *as-1* (−520 bp), that recruit the TFs and induce the expression of the *VvPR1* gene. This study expanded the available information regarding SA-induced defense in susceptible grapes and recognized the molecular mechanisms through which this defense might be mediated.

## Introduction

Grapevine (*Vitis* spp., family Vitaceae) is a commercially important fruit grown around the globe, and its history extends over 8,000 years (Fischer et al., 2004). Currently, most worldwide production is from the European grapevine (*V. vinifera* L.), which is the main table grape species in China. Because of the character of the East Asiatic climate (monsoon) with high precipitation and temperature, grapes are vulnerable to different fungal diseases (Wan et al., 2015; Pap et al., 2016; Sapkota et al., 2019). A major fungal disease affecting grapevine is grape white rot (caused by *Coniella diplodiella* (Speg.) Sacc.), which reduced the grape yield by at least 16.3% in grape-producing regions (Li et al., 2008). White rot disease infects the leaves, berries, and new shoots. The application of antifungal agents is recommended for successful grape production, but those agents have hazardous effects on the environment. At present, many grapevine cultivars are found resistant to grape white rot, especially the wild grape relative, making it an important source for grape white resistance through breeding.

Plants have sophisticated defense mechanisms for pathogen recognition. In the systemic acquired resistance (SAR) pattern recognition receptor (PRR)-triggered immunity, PTI is the first tier of plant immunity. PTI is governed by the recognition of pathogen-associated molecular patterns (PAMPs) and is very effective against most pathogens (Boller and Felix, 2009; Dodds and Rathjen, 2010; Dou and Zhou, 2012). However, to overcome the PTI, pathogens manufacture effector proteins and penetrate them in into the host cell to increase their survival in the host. Then, the plant mediates its second tier of immunity, effector-triggered immunity (ETI), to respond to the effector-triggered susceptibility through their resistance genes by recognizing pathogen effectors (van Loon et al., 2006; Wang et al., 2012; Sawinski et al., 2013). Pathogenesis-related proteins (PRs) are activated by abiotic and biotic factors and play vital roles in plant defense (Sels et al., 2008); the expression of PRs can increase plant resistance following infection by pathogenic bacteria (Wildermuth et al., 2001). A major protein in the PR family is PR1, that responds to disease resistance in plants against the environmental stress (Godiard et al., 1990). Jasmonic acid (JA) and SA perform a vital role in plant SAR through PR1 proteins, different transcription factors, and enzymes that control the expression of PR1 in these pathways (Després et al., 2000; Gu et al., 2002; Després et al., 2003; Lorenzo et al., 2004). It was also found that methyl jasmonate (MeJA) induced the defense genes expression through the MAPK pathway in grapevine (Rahman et al., 2022). PR1 proteins are considered to be antioomycete and antifungal proteins, and the function of PR1 is still enigmatic, in contrast to other PR proteins whose functions have been elucidated (Joshi et al., 2021; Kaur et al., 2022). Recently, tomato and tobacco PR1 protein sterol-binding activity has been found critical for its antimicrobial activity (Gamir et al., 2017). Plant resistance against pathogenic oomycetes and bacteria in tobacco has been found higher from the overexpression of the pepper basic PR1 gene (Sarowar et al., 2005). The promoters of pathogenesis-related genes contained some cis-regulatory elements responsible for their expression upon phytohormones signaling. For example, there are some SA-inducible cis-regulatory elements in promoters of some pathogenesis-related genes in *Arabidopsis thaliana* PR-1 and tobacco PR-1a, like *activation sequence-1* (as-1). In the promoter of PR-1a, tobacco possesses an as-1-like element with inverted TGACG motifs, a binding site for TGA transcription factors (Strompen et al., 1998; Niggeweg et al., 2000; Grüner et al., 2003). The promoters of the 39 BR signaling genes are involved in various regulatory mechanisms and interdependent processes that influence growth, development, and stress response in rice (Ahmar and Gruszka, 2022).

SA is a well-studied phenolic plant hormone that plays an important role in plant defense against the hemi-biothrophic and biothrophic pathogens that depend on the living cells of host for the successful infection. Neurotransmitter interactions mediate antioxidant defenses under induced oxidative stress in plants (Vlot et al., 2009; Raza et al., 2022). SA biosynthesis may activate the resistance genes that initiate SAR through hypersensitive response by accumulating at the place of pathogen attack. They are then distributed to other plant parts as a mobile signal in the form of methyl salicylate to induce the defense response (Zhao et al., 2005). Many plants cannot effectively employ these mechanisms. It was found that exogenous SA application has induced PR proteins (Raskin, 1992), as SA effectiveness has been verified against bacteria (Mohan Babu et al., 2003; Mandal et al., 2013), fungi (He and Wolyn, 2005; Mandal et al., 2009), and viruses (Van Huijsduijnen et al., 1986). SA treatment in tobacco reduced the multiplication of bacteria and disease symptoms against *Erwinia carotovora* (Palva et al., 1994). The exogenous treatment of SA enhances the resistance of asparagus against *Fusarium oxysporum* f. sp. asparagi, with increases in the levels of lignifications, phenylalanine ammonia-lyase, and peroxidases (He and Wolyn, 2005). Similarly, in tomato roots, SA application induced the defense response against the *F. oxysporum* f. sp. Lycopersici and reduced vascular browning symptoms by increasing phenylalanine ammonia-lyase, peroxidases, β-1,3-glucanase, and lignifications (He and Wolyn, 2005; Mandal et al., 2009). PR proteins were also induced by the SA application in grapevine leaves [10]. The PR1 expression induced through exogenous application of SA against the alfalfa mosaic virus (A1MV) (Van Huijsduijnen et al., 1986). The SA master regulator NONEXPRESSOR OF PATHOGENESIS-RELATED GENES 1 (NPR1), degraded from oligomer to monomer with the elevation of SA accumulation in the infected cell through NPR3 and NPR4, which results in the effector-triggered cell death; NPR1 also mediated SA resistance in the neighboring cells to promote cell survival (Fu and Dong, 2013; Yan and Dong, 2014). In addition, SA signaling engages a feedback circuit to amplify defense responses that are negatively regulated by EDR1, a MAPKKK (Frye et al., 2001; Xiao et al., 2005).

Previously, researchers analyzed the individual mechanisms for the evaluation of induced resistance involved in stress response. However, these techniques provide limited explanations for the defense mechanisms promoted by the elicitors. To evaluate the elicitors in plant defense, a large number of studies have been conducted on gene expression in response to different phytohormones. A transcriptome study performed on sorghum with exogenous SA treatment induced numerous defense genes, such as several PR genes, the JA pathway, and phenylpropanoid, that exhibit different pattern of synergistic as well as antagonistic effects between SA and JA. Exogenous SA application of on grapevine leaves induced different PR proteins (Renault et al., 1996), but no transcriptome study has been reported on the grapevine leaves in SA application. Our study compared the pathogenesis of grape white rot disease in *V. vinifera* L. cv. Zaotianmeiguixiang and Chinese wild grape species *V. davidii,* in which the wild grape cultivar showed resistance against white rot with higher levels of SA. Transcriptome analysis was performed on susceptible grapevine leaves against the SA treatment to investigate the important transcripts responsible for the defense response. This study was proposed to investigate the effect of exogenous SA application on the susceptible grapevine cultivar through the induction of SA marker genes by transcriptome analysis. For further functional analysis, a candidate gene, PR1, was selected for promoter analysis against the SA treatment through transient expression on tobacco leaves.

## Materials and methods

### Plant material

Grapevine 2-year-old “resistant” *V. davidii* accession 0940 and “susceptible” *V. vinifera* (Zaotianmeiguixiang) plants were grown in a greenhouse under controlled conditions (25 ± 5°C, 16-hour light/8-hour dark photoperiod, 65% relative humidity) at the Zhengzhou Fruit Research Institute, Chinese Academy of Agricultural Sciences (CAAS), Henan, China. Sand and peat (50/50, v/v) used as potting media, and the plants were watered twice in a week. Tobacco (*Nicotiana benthamiana*) plants were grown under greenhouse conditions (16-hour light/8-hour dark photoperiod, 65% relative humidity, 25 ± 5 °C) in potting media (vermiculite/perlite/moss, 2/3/5, v/v/v). The *N. benthamiana* plants were used for the *Agrobacterium*-mediated transient assay at the sixth-leaf stage.

### Pathogenicity test and SA treatment

For the pathogenicity test, the causal organism of white rot (*C. diplodiella,* strain WR01) was taken from the Institute of Plant Protection, CAAS, and grown at 28°C on potato dextrose agar medium. The plants were inoculated with four mycelium gelose discs (diameter = 2 mm) of *C. diplodiella* on each leaf using small pins, and the leaf was covered with a plastic bag to retain the moisture throughout the infection period. After 72 h of post-inoculation (hpi), leaf samples were observed. Each treatment had three control replicates and four independent biological infected replicates. The susceptible *V. vinifera* (Zaotianmeiguixiang) plants were sprayed with 100 μM SA (Sigma-Aldrich Chemicals GmbH, Schnelldorf, Germany) solution containing 0.05% (v/v) Tween 20 treatment, and control plants were sprayed with 0.05% (v/v) Tween 20 (Yang et al., 2019). Three replications were taken for each treatment; each replication contained three leaf samples, and leaf samples were collected after 12, 24, and 48 h of treatment and immediately stored at −80°C before RNA extraction.

### Salicylic acid measurement

Grapevine leaves were collected after 12, 48, and 72 h of after the white rot (*C. diplodiella,* strain WR01) inoculation and kept in liquid nitrogen. SA was measured according to the procedure detailed in Hu et al. (2019). A triple-quadrupole LC/MS system (1290 Infinity II-6470, Agilent Technologies, USA) was used for the measurement of SA content, as explained by Hu et al. (2019). Three independent replicates were used for this experiment.

### Total RNA extraction, library construction of mRNA, and data analysis

The total RNA was extracted following the CTAB-pBIOZOL reagent and ethanol precipitation protocol recommended by the manufacturer (Mu et al., 2017). The mRNA was purified using oligo(dt) attached to magnetic beads. The mRNA was fragmented into small fragments with the fragment buffer at the appropriate temperature. The first strand of cDNA was synthesized through reverse transcription with a random hexamer primer; then second-strand cDNA was synthesized. A-Tailing Mix and index adapters were added to the mixture for end repairing of RNA. Previously synthesized cDNA fragments were amplified through PCR, purified by Ampure XP beads, and dissolved in the EB solution. The quality control of the product was validated on the Agilent Technologies 2100 bioanalyzer. For the final library, the double-stranded PCR product was denatured through heating and circularized by the splint oligo sequence. The single strand circular DNA (ssDNA) was considered as the final library. The final library was amplified with phi29 to make DNA nanoball (DNB), which had 300 copies of one molecule. DNBs were loaded into the patterned nanoarray, and pair end 100 bases reads were produced on DNBSeq platform (BGI-Shenzhen, China). This project used SOAPnuke (v1.4.0), the filtering software that was independently developed by BGI. First, the reads containing the unknown base N content greater than 5% and the reads containing the connector (connector contamination) were removed. The low-quality reads (those with a quality value of less than 15, which account for more than 20% of the total number of bases in the reads) were also removed. The filtered “clean reads” are saved in FASTQ format. To compare the RNA-seq reads with the reference genome of *Vitis vinifera* (http://plants.ensembl.org/Vitis_vinifera/Info/Index?db=core;g=VIT_08s0007g00570;r=8:1482803614830056;t=VIT_08s0007g00570.t01) (accessed on 10 January 2022), hierarchical indexing for spliced alignment of transcripts (HISAT) (v2.1.0) (Kim et al., 2015) software was used. Bowtie2 (v2.2.5) was used to calculate the mapping rate to align clean reads to the reference gene sequence, and RSEM was used to calculate the expression levels of genes and transcripts (Li and Dewey, 2011; Langmead and Salzberg, 2012). Bioinformatics analysis was performed on the successfully mapped clean reads on the reference genome.

### Quantitative real-time PCR

A Roche Light Cycler 480 Real-Time PCR system and a Roche Light Cycler 480 SYBR Green I Master were used to run the qRT-PCR. The qRT-PCR conditions were as follows: 95°C for 30 s for denaturation, followed by 40 cycles of 5 s at 95°C, at 55°C for 30 s, and 72°C for 10 s with the primers (Supplementary Table S1). Three biological replicates were used for all of the reactions, and Bio-Rad CFX Manager software was used to determine the threshold cycle (Ct). The qRT-PCR method was used according to the manufacturer’s instructions. The relative quantitative expression level was calculated by the 2−∆∆CT method (Livak and Schmittgen, 2001). The gene expression level of grapevine level was analyzed using *VvActin* as the reference gene*.*

### Promoter isolation of *VvPR1* gene and sequence analysis

The genomic DNA was extracted from the grapevine leaves using a DN 15-Plant DNA Mini Kit according to the manufacturer’s instructions. The DNA concentration was measured using a NanoDrop 1000 spectrophotometer (Thermo Scientific, Waltham, MA, USA). The primer pair for the promoter of *VvPR1* (VIT_03s0088g00810) was designed from the reference sequence of *V. vinifera* (Supplementary Table S2). A region approximately 1900 base pairs upstream from the coding region was thought to be the putative *VvPR1* promoter. The *VvPR1* promoter was amplified using the Premix high-fidelity (Takara) enzyme, and the PCR conditions were followed according to Rahman et al. (2022). The PCR product was purified on 1.5% agarose gel, cloned on the *pCE2* Blunt vector, and sequenced for the verification of the promoter sequence. The PlantCARE online tool was used to predict the cis regulatory elements in the *VvPR1* promoter (http://bioinformatics.psb.ugent.be/webtools/plantcare/html/) (accessed on 17 February 2022) (Lescot et al., 2002).

### Construction of beta-glucuronidase vectors

**FIGURE 1:**
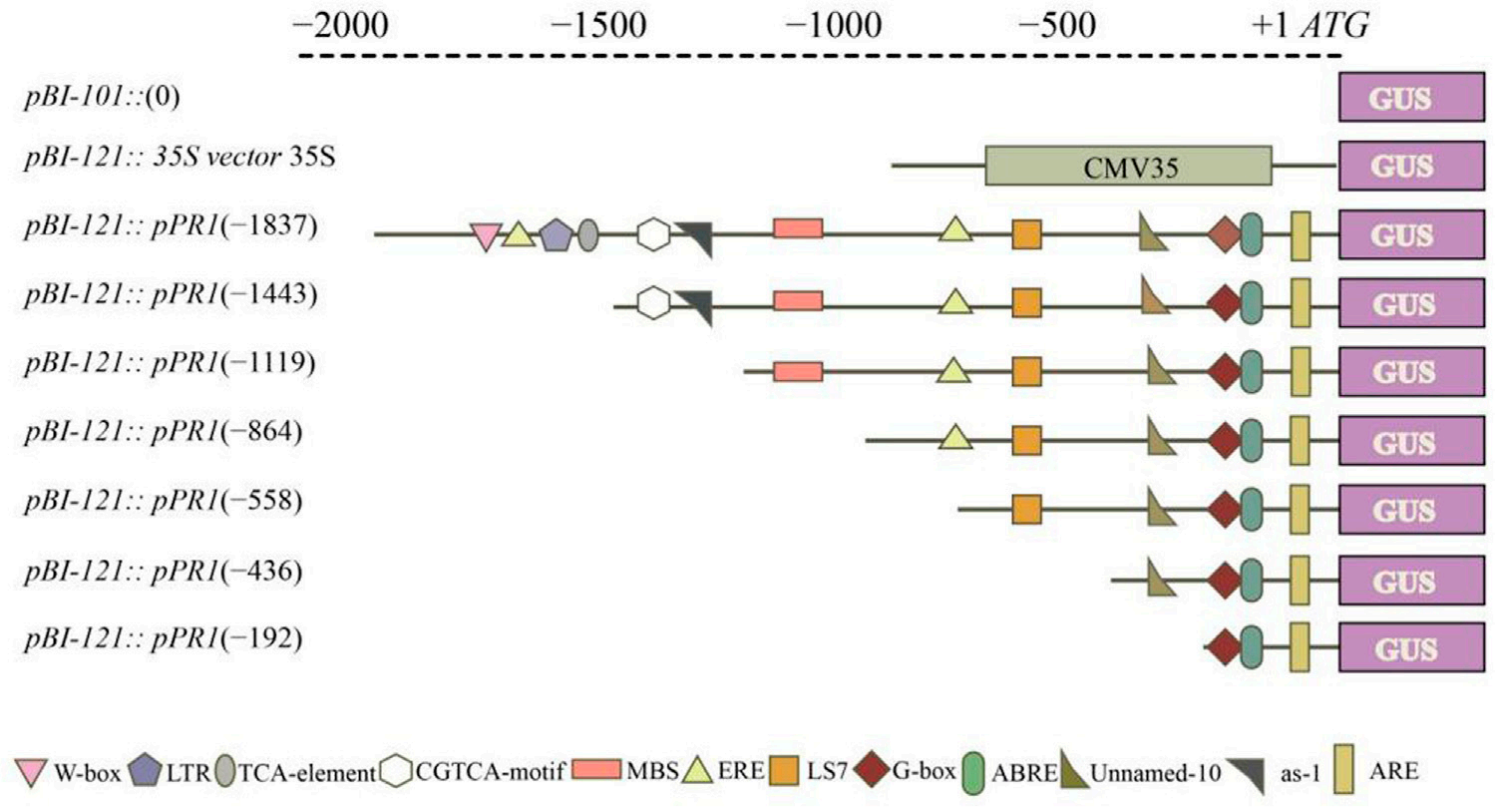
Schematic representation of the *VvPR1* promoter. Constructs for assaying GUS (β-glucuronidase) expression in tobacco leaves. The constructs of serially deleted promoter fragments of the *VdPR1* gene were fused to the GUS reporter gene in the vector *pBI-121*.

The *VvPR1* promoter serially deleted into the promoter fragments and cloned them into the *pCE2* Blunt vector by designing primers of different lengths from promoter sequence (Supplemetary Table S2). Each forward primer contained the HindIII restriction site at the 5′ end, and the reverse primer contained the BamHI restriction site at the 5′ end. The PCR reaction was performed, and the PCR product was purified on the agarose gel using the gel extraction kit. Meanwhile, the expression vector (*pBI-121*) was also digested by restriction enzymes (HindIII and BamHI) for 2 hours and then subcloned with the purified PCR products. Seven promoter fragments (−1837 bp to ATG, −1,443 bp to ATG, −1,119 bp to ATG, −864 bp to ATG, −558 bp to ATG, −436 bp to ATG, and −192 bp to ATG) were separately fused into the expression vector *pBI-121* with the GUS reporter gene, yielding *pBI-121*:*pPR1* (Figure 1). The strong promoter CaMV35 in the expression vector *pBI-121* was used as positive control, and *pBI-101* with no promoter was used as a negative control. All recombinant vectors were cloned and propagated in *Escherichia coli* (DH5α strain). Then, the constructs of promoter/GUS fusion were inserted into the *Agrobacterium tumefaciens* strain GV3101 by heat shocks.

### *Agrobacterium*-mediated transient expression assay with abiotic stress treatment

*Agrobacterium* was used for the transient expression, as mentioned by Yang et al. (2000). The serially deleted fragments of promoter:GUS harbored by *Agrobacterium* GV3101 were grown in the LB medium supplemented with the antibiotics rifampicin (60 μg ml^−1^) and kanamycin (50 μg ml^−1^). The *Agrobacterium* strains were cultured in 50 ml of LB broth at 28 °C overnight. The LB broth was centrifuged for 10 min at 6000× g to collect the *Agrobacterium* cells, which were then resuspended in infiltration media (10 mM MgCl_2_, 100 µM acetosyringone 10 mM MES, (pH 5.6), (Sigma-Aldrich)) and adjusted to an OD600 of 0.8. A needleless syringe was used to inject the infiltrate of *Agrobacterium* suspension into tobacco leaves, which were then placed in a moist chamber at 26°C for 24 h and then shifted to the growth room. SA (100 μM, 0.2% Tween-20) and ABA (100 μM, 0.2% Tween-20) treatment was applied to the tobacco leaves harboring the *pBI-121*:*pPR1*/GUS, and the control plants were only sprayed with 0.2% Tween-20. SA-treated and control plants were placed in the baskets and covered with the polyethylene-perforated plastic bags (having six holes (1 cm diameter) on each side and 0.03 mm thickness). Three replications were performed for each treatment, and each was also repeated three times for the transient GUS expression on the tobacco leaves. Samples were collected after 24 h of treatment.

### GUS activity measurement

Histochemical staining analysis was performed to measure the GUS expression transiently as described by Jefferson et al. (1987). GUS staining solution was prepared as explained by Yu et al. [80]. Tobacco leaf discs were collected and dipped in a GUS staining solution (0.5 mg L−1 5-bromo-4-chloro-3-indolyl-b-D- glucuronic acid, 10 mM Na_2_EDTA, 100 mM NaH_2_PO_4_, 0.1% Triton X-100, and 0.5 Mm K_4_Fe(CN)_6_.3H_2_O (X-Gluc, Sigma-Aldrich, Shanghai, China), pH 7.0) and incubated at 37°C for 24 h. Leaf discs were incubated in 70% ethanol at 37°C to remove the chlorophyll contents for more clear observation and rinsed several times with ethanol. The quantitative GUS assay of promoter transiently expressed in tobacco leaves was measured as described by Jefferson et al. (1987). Microtubes were filled with leaf powder after grinding. The extraction buffer, phosphoric acid buffer (0.5M EDTA, TritonX-100(10%), 2M KPO_4_ (pH 7.8)), 80% glycerol, and beta mercapto ethanol (1 ml), was added to the microtube and vortexed. The material inside the microcentrifuge tube was centrifuged at 120,00× g for 15 min at 4°C, and the supernatant was transferred to a microcentrifuge tube already placed on ice. The whole fluorogenic reaction was performed at a volume of 1 ml mixed with extraction buffer in 1 mM 4-methylumbelliferyl-h-D-glucuronide (MUG) (Duchefa Biochemie, Haarlem, Netherlands), which also comprised an aliquot of protein extract at a volume of 0.1 ml. The standards of bovine serum albumin (BSA) were used for the quantification of protein extracts as explained by Bradford (1976). The results were found to be similar after measuring GUS three times.

### Statistical analysis

Microsoft Excel (2016) was used for values calculation, and Student’s t-test was used for calculating differences among values. The significance levels are shown as follows: * represents *p* ≤ 0.05, and ** represents *p* ≤ 0.01. All experiments were repeated three times with three independent biological replicates.

## Results

### Structure and disease symptoms of grapevine leaves

**FIGURE 2:**
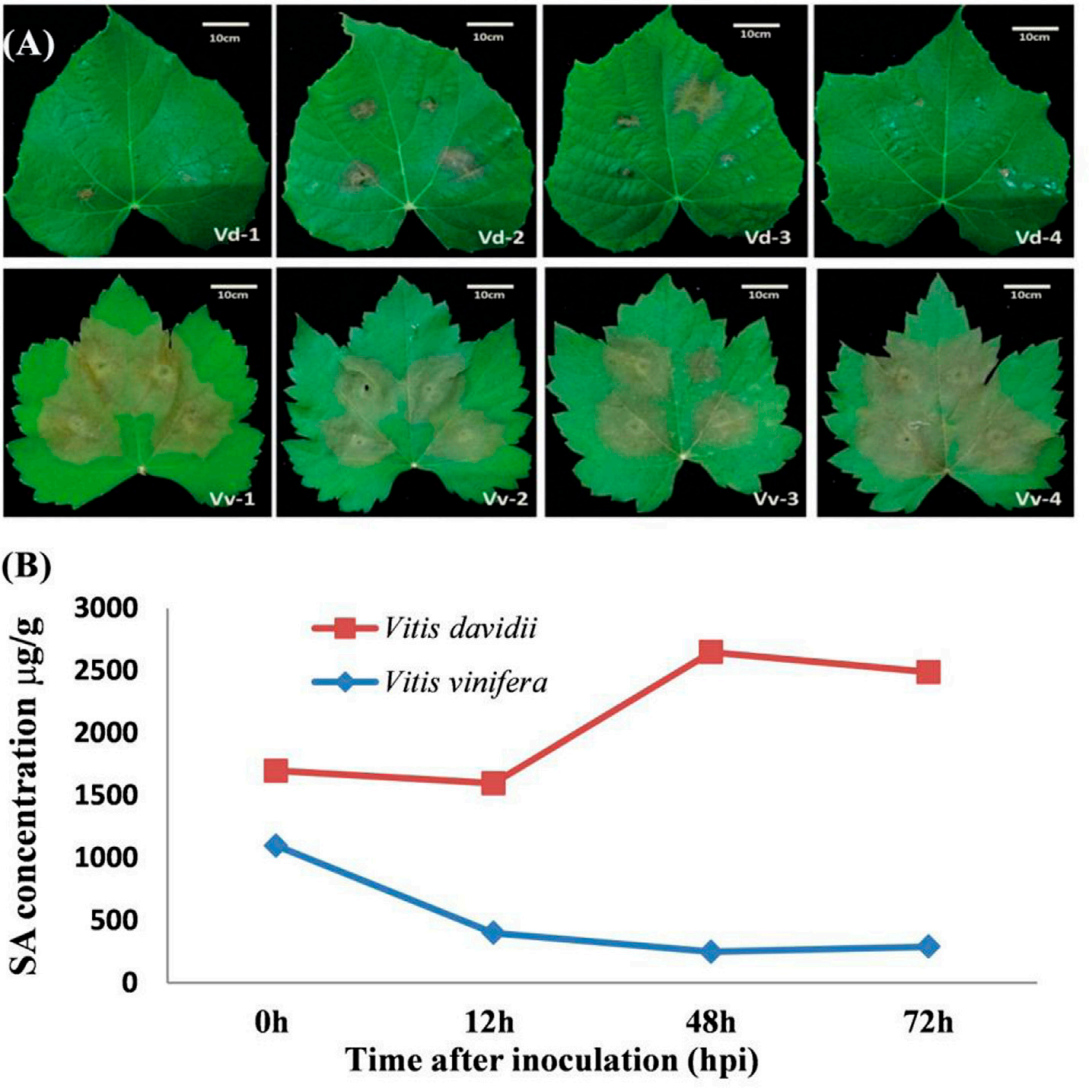
**(A)** Symptoms of *Coniella diplodiella* infection on the leaf samples of *Vitis vinifera* Manicure Finger (Vv) and *Vitis davidii* accession 0940 (Vd). Two-week-old leaf samples were collected at the 3-4 position. Typical hypersensitive response (HR) symptoms were observed in Vd but not in Vv at 72 h post-infection (hpi). There were four replications of each species. **(B)** Endogenous measurement of SA from the *V. davidii* and *V. vinifera* after 0, 12, 48, and 72 h of white rot disease (*Coniella diplodiella*) inoculation.

In this study, the grapevine leaves were inoculated with *C. diplodiella,* and disease symptoms were found to be higher in *V. vinifera* than the *V. davidii* after 72 h of inoculation (Figure 2A). The hypersensitive response (HR) occurred in *V. davidii,* where the sudden cell death happened at the site of infection and stopped the spreading of the pathogen infection. At the base of the leaf structure, there was no significant difference in leaf thickness between *V. davidii* and *V. vinifera* (Table 1). The endogenous SA contents were also measured after *C. diplodiella* inoculation, and SA contents were found to be higher in *V. davidii* than *V. vinifera* after specific time points of inoculation (Figure 2B).

**TABLE 1:** Comparison of two *Vitis* species after *Coniella diplodiella* inoculation.

Species	Diameter (mm)	Leaf thickness (mm)	Rate of incidence (%)	Disease index	Disease index (%)	Disease rank
*Vitis davidii*	8.24 ± 5.02^a^	0.081 ± 0.006^a^	75.00	0.08	8.33	HR
*Vitis vinifera*	34.03 ± 4.36^b^	0.113 ± 0.002^a^	100.00	0.78	77.78	HS

Data are the mean ± SD, n = 04, and significant differences were assessed using analysis of variance.

a,b represents the significant difference between the means, and means sharing similar letter in a column are statistically non-significant (*p* > 0.05).

### Transcriptomic analysis of SA-treated grapevine leaves at different time points

**FIGURE 3:**
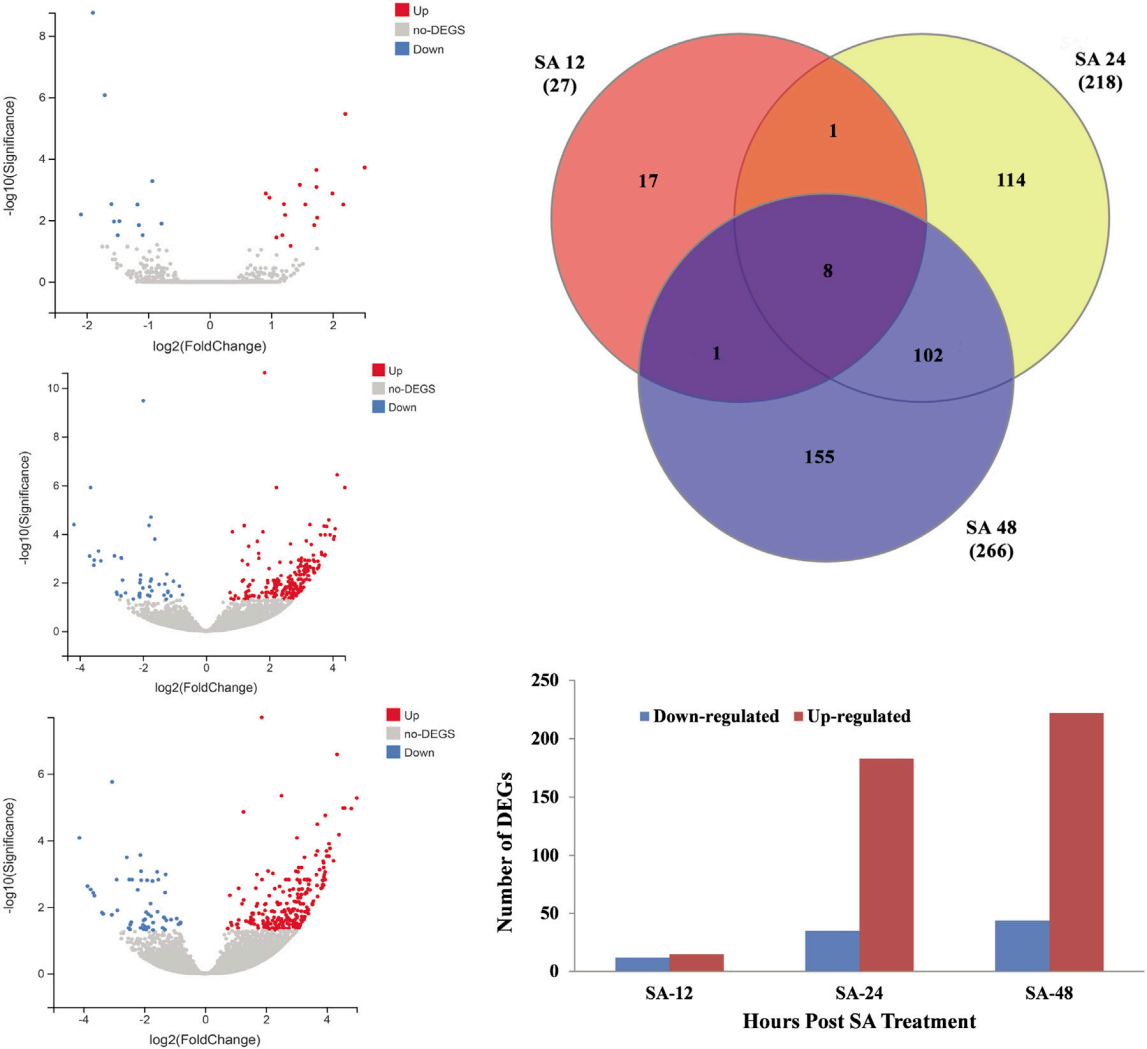
Distribution of DEGs from RNA-seq data. **(A)** Volcano graph of DEGs representing the downregulated genes with blue color and upregulated DEGs with red color after 12 h of SA treatment. **(B)** Volcano graph of DEGs representing the downregulated genes with blue color and upregulated DEGs with red color after 24 h of SA treatment. **(C)** Volcano graph of DEGs representing the downregulated genes with blue color and upregulated DEGs with red color after 48 h of SA treatment. **(D)** Venn diagram analysis of DEGs identified from all time points. **(E)** Total number of DEGs that were significantly down- or upregulated in response to SA treatment. Log2 FC ≥ 1 or ≤−1 and *p* < 0.01 FDR.

Transcriptome analysis was performed on the grapevine leaves treated with SA after 12 h and 48 h. As most disease attack happened on the aerial parts of the plants, SA treatment was applied to grapevines. An individual leaf sample comprised ≥6.3 Gb data with a Q20 ≥ 97.14% quality score. A total of 57.6 Gb clean data were mapped from twelve leaf samples (Table 2). On average, from each leaf sample, 88.17%–89.72% reads were uniquely mapped and aligned with reference genome *V. vinifera* L. The expression levels (*p* ≤ 0.05) of the control and the SA-treated samples were compared on the basis of a Cuffdiff analysis. For differentially expressed genes (DEGs), *p*-values (0.01) and log_2_-fold changes (log_2_FC) ≥ 1 or ≤−1 were used for threshold values after 12 h and 24 h of SA treatment. To identify the DEGs against the SA application after 12, 24, and 48 h, a volcano plot was designed against FC(log_2_) and −log10(significance) (Figures 3A–C). A Venn diagram was used to show the distribution and representation of DEGs after 12h and 48 h of SA treatment. The Venn diagram represents that 17, 114, and 155 genes were the unique set of genes that were expressed after 12, 24, and 48 h of SA treatment, respectively, and eight genes were coregulated at all time points (Figure 3D). A total of 511 DEGs were identified in which 12, 33, and 44 genes were downregulated, and 15, 183, and 240 genes were upregulated after 12, 24, and 48 h of SA treatments, respectively (Figure 3E).

**TABLE 2:** Transcriptome raw data and differentially expressed genes.

Sample	Total raw reads (M)	Total clean reads (M)	Clean reads Q20 (%)	Clean reads ratio (%)	Total mapping (%)	Transcripts with changed expression
CR	44.40	42.46	97.07	95.63	88.17	
SA12	43.82	42.42	97.16	96.81	89.72	27
SA24	43.82	42.34	97.08	96.62	89.52	218
SA48	44.40	42.66	97.19	96.07	89.23	266
Total	307.91	297.42	679.95	676.16	619.66	511

All DEGs (down- and upregulated) were obtained from the transcriptome data after SA treatment and compared with controls according to the Cuffdiff analysis.

### Gene ontology analysis

Gene ontology (GO) analysis of the DEGs after 12, 24, and 48 h of SA treatment was performed to classify them into three main domains of GO: biological process, cellular component, and molecular function. The biological process category contained nine GO terms, of which cellular process contained a significant number of enriched genes, followed by metabolic process. The cellular component contains three biological terms in which a cellular anatomical entity was enriched with significant DEGs. The molecular function has seven GO terms in which DEGs were significantly enriched in catalytic activity followed by binding (Supplementary Figure S1). More DEGs were enriched in the GO terms and domains after 48 of SA treatment. Three domains from three GO terms (cellular process, cellular anatomic entity, and catalytic activity) had the maximum number of downregulated genes (14, 32, and 25, respectively) and the maximum number of upregulated genes (83, 131, and 127, respectively) genes after the 48 h of SA treatment (Table 3).

**TABLE 3:** Functional categorization of DEGs through GO analysis after SA treatment with different time points.

	Name	SA 12		SA 24		SA 48	
		Down	Up	Down	Up	Down	Up
Biological process	Biological regulation	0	2	5	28	10	30
	Cellular process	0	5	15	68	18	79
	Developmental process	0	1	1	9	3	11
	Immune system process	0	2	2	2	0	4
	Interspecies interaction between organisms	0	0	2	3	0	5
	Localization	0	0	7	17	7	15
	Metabolic process	5	4	12	63	16	80
	Multi-organism process	1	1	0	2	0	2
	Multicellular organismal process	0	0	2	10	1	11
	Reproductive process	0	0	0	4	0	4
	Response to stimulus	2	3	7	29	10	37
	Signaling	0	1	0	18	1	22
Cellular component	Cellular anatomical entity	4	6	28	124	33	130
	Intracellular	1	2	12	44	15	49
	Protein-containing complex	0	1	2	12	0	8
Molecular function	Antioxidant activity	0	0	0	1	0	5
	Binding	2	5	16	108	21	112
	Catalytic activity	5	7	20	113	27	125
	Molecular function regularity	0	0	2	2	1	6
	Molecular transducer activity	0	1	0	0	0	7
	Structural molecule activity	0	1	0	1	0	3
	Transcription regulator activity	0	1	0	3	0	5
	Transporter activity	0	0	4	14	4	11

Individual category of GO term may have more one gene products.

### Kyoto Encyclopedia of Genes and Genomes analysis

**FIGURE 4:**
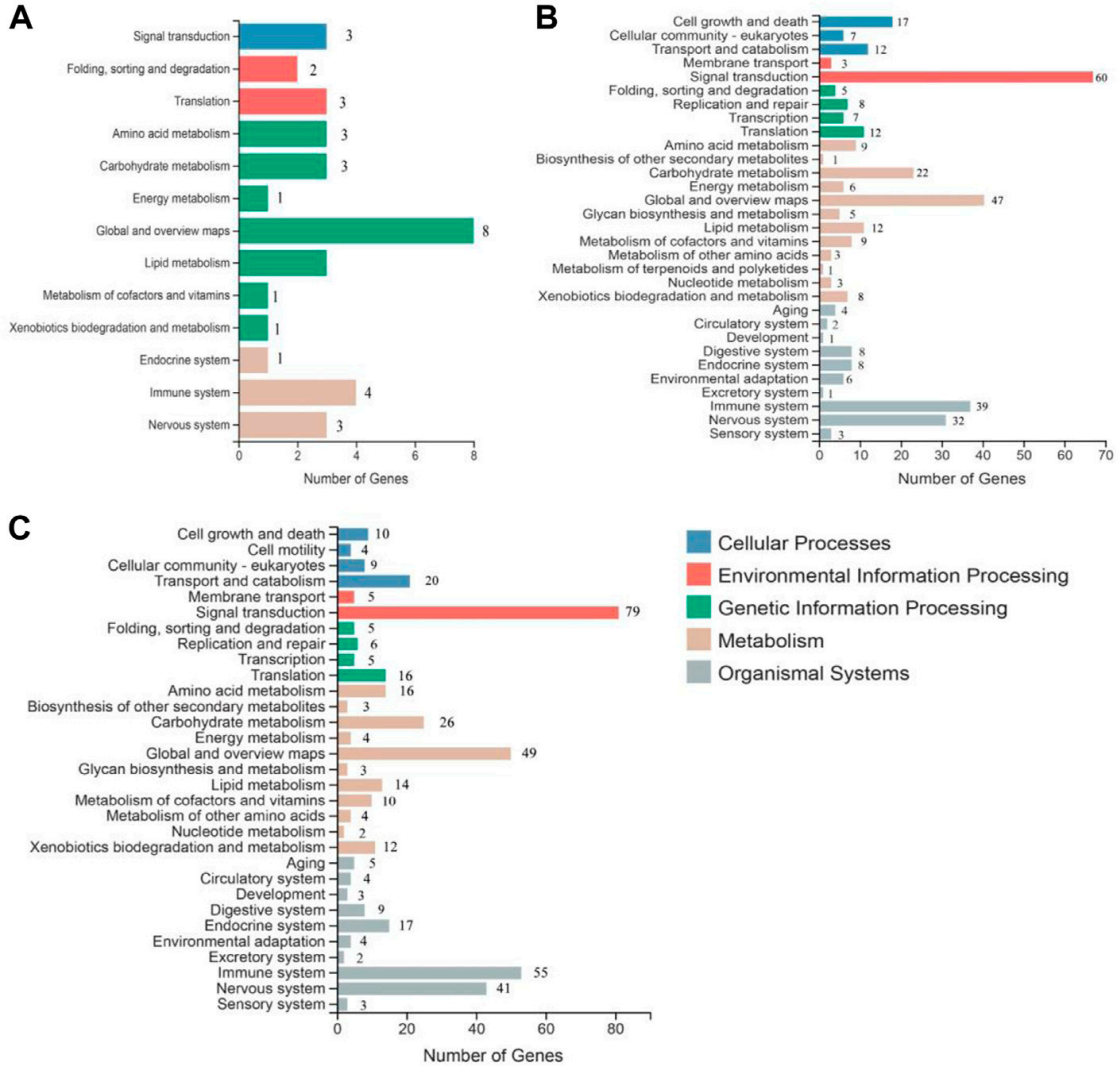
Kyoto Encyclopedia of Genes and Genomes (KEGG) analysis of significantly expressed transcripts at all time points of SA treatment: **(A)** 12 h of SA treatment, **(B)** 24 h SA treatment, and **(C)** 48 h of SA treatment.

To understand the biological pathways induced by SA in the grapevine leaves, all DEGs were mapped against the Kyoto Encyclopedia of Genes and Genomes (KEGG) database. DEGs mapped on the KEGG database revealed that most significant changes in response to SA treatment were related to the plant immune and defense response. According to KEGG, the metabolic pathway divided into five categories: environmental information processing, cellular processes, organic systems, genetic information processing, and metabolism. The highest number of KEGG pathways induced in grapevine leave were global and overview map (8, 47, and 49), signal transduction (3, 60, and 79), and immune system (4, 39, and 55) after 12, 24, and 48 h of SA treatment (Figure 4). The top 20 KEGG pathways comprised most of the defense and immune related pathways; among them, phenylalanine metabolism, MAPK signaling pathway, Ras signaling pathway, alanine aspartate, glutamate metabolism, and Toll-like receptor signaling pathway that have a crucial role in plant disease resistance (Supplementary Figure S2).

### SA plant defense signaling

**FIGURE 5:**
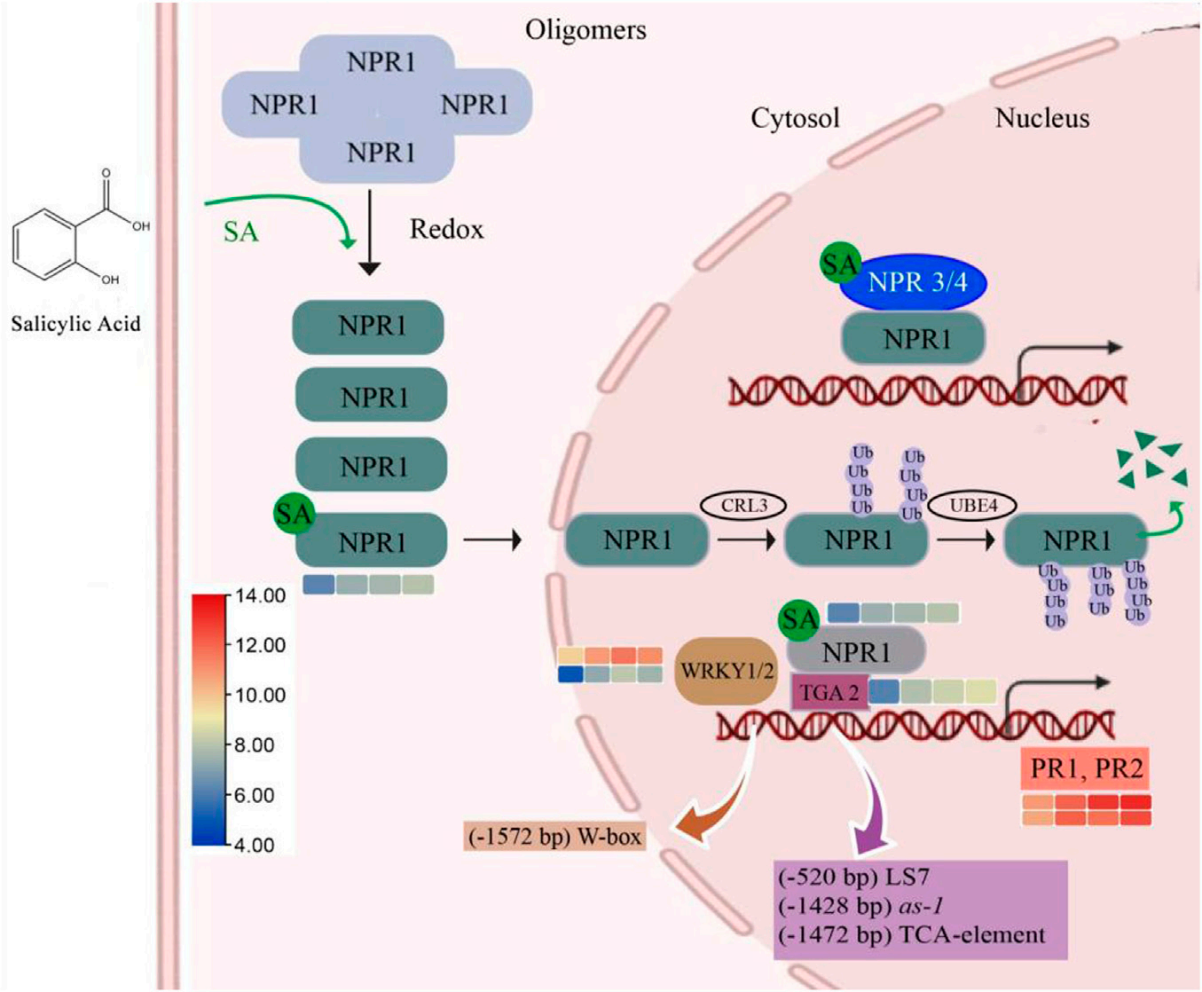
SA pathway related to plant defense. A higher SA level can induce the monomerization process of NPR1 and induced NPR1-dependent gene expression through direct interactions with TGA transcription factors. Meanwhile, direct binding with SA derepressed the suppression of NPR3 and NPR4 on SA-induced genes, which further enhanced SA-induced NPR1-dependent gene expression. Efficient turnover of monomeric NPR1 proteins in the nucleus is required for a rate-limited SA-induced gene expression, and this is also dependent on the homeostasis of NPR1-ubiquitination.

In plant defense, SA helps to encode the proteins related to antimicrobial activities through the induction of pathogenesis-related (PR) genes. To date, 17 PR families have been identified; among them, PR1, PR2, and PR5 are induced by biotrophic and semibiotrophic pathogens. Additionally, the expression of *VvPR1* and *VvPR2* was upregulated (1.14 to 2.44 fold) by the exogenous application of SA on grapevine leave after different time points (12, 24, and 48). A master regulator of SA-mediated plant defense is *VvNPR1* (non-expresser of PR genes 1), and it regulates the *VvPR1* gene through the binding with TGA transcription factors. *VvNPR1* was upregulated (1.16 to 1.97 fold) at all time points after SA treatment, and *VvTGA2* was also identified from the transcriptome data and upregulated (1.74 to 2.59 fold). *VvWRKY1* and *VvWRKY2,* another transcription factor, was also upregulated (1.15 to 3.16 fold) after the 12, 24, and 48 h SA treatment on the grapevine leaves (Table 4; Figure 5).

**TABLE 4:** DEGs involved in SA plant defense pathway after 12, 24, and 48 h of SA treatment.

Gene description	Gene ID	Log2-fold change		
		SA 12	SA 24	SA 48
NPR1	VIT_11s0016g01990	1.16	1.71	1.47
PR1	VIT_03s0088g00810	1.23	2.19	2.44
PR2	VIT_08s0007g06060	1.52	1.14	1.97
WRKY1	VIT_17s0000g01280	1.15	1.96	1.41
WRKY2	VIT_01s0011g00220	2.29	3.16	2.63
TGA2	VIT_08s0007g05170	1.74	2.25	2.59

### Verification of differential gene expression

**FIGURE 6:**
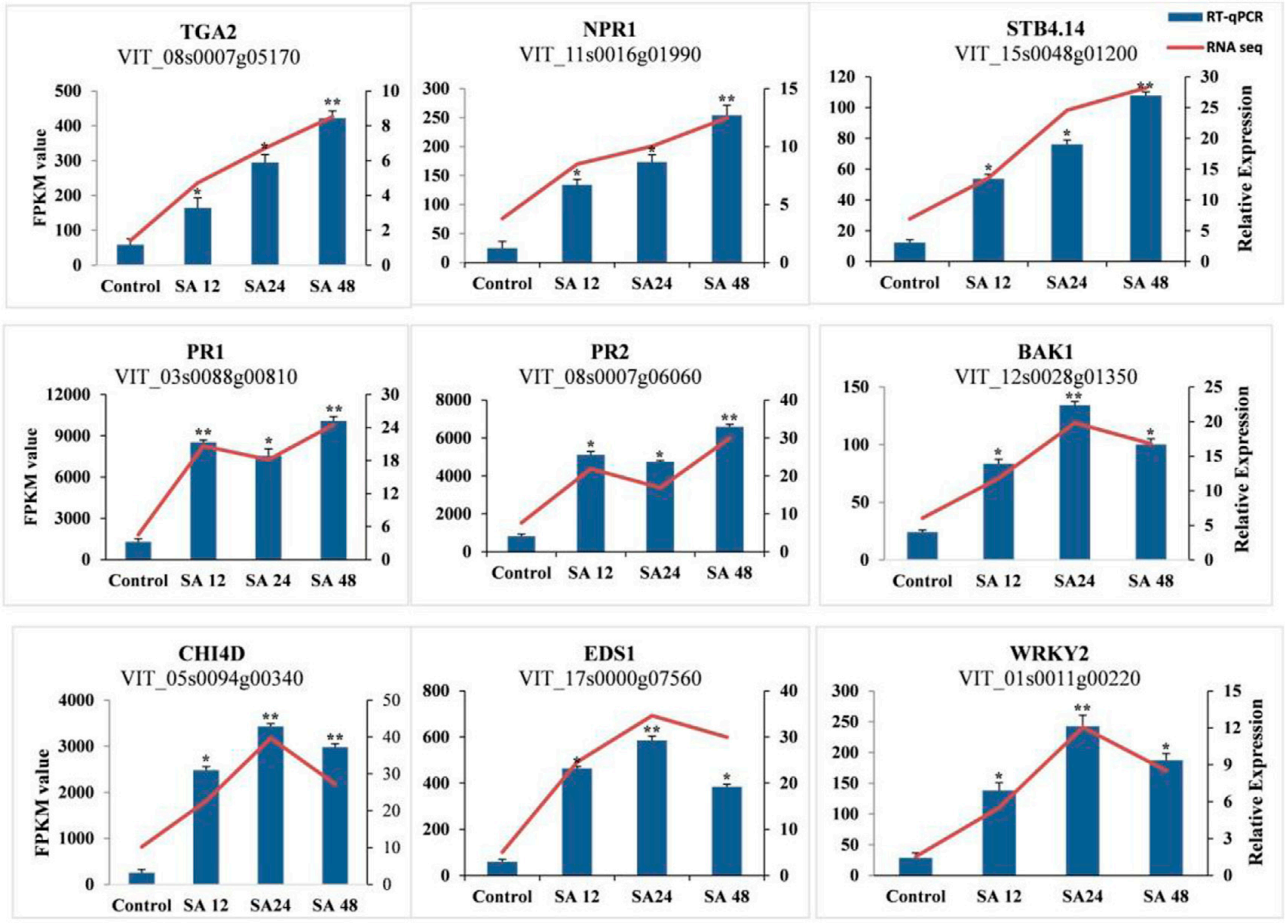
Validation of RNA-seq data with RT-qPCR. RT-qPCR of the selected DEGs was used for the verification of RNA-seq data. Error bars indicate the standard error as mean +SD. Significant differences between the control and treated samples are indicated by an asterisk (*). The sign * represents p ≤ 0.05, and ** represents p ≤ 0.01.

The validation of the RNA-seq data was performed by selecting random transcripts from significantly expressed transcripts. We selected only nine genes for qPCR, of which five genes (*VvPR1, VvPR2, VvTGA2, VvSTB-14*, and *VvNPR1)* were continuously increasing their expression from all time points. The expression of *VvWRKY2, VvEDS1,* and *VvCHI4D* increased at 24 h of SA treatment, whereas the expression of *VvBAK1* and *VvTGA2* decreased at 24 h of SA treatment. The qPCR expression results were found consistent with transcriptome data (Figure 6). The most important plant defense gene, *VvPR1,* is a DEG that responds to different biotic and abiotic stress conditions, so this gene was selected for further functional validation in response to SA application.

### *VvPR1* promoter sequence analysis

About 1837 bp of *VvPR1* upstream from the ATG was considered as a putative promoter and cloned in a *pCE2* Blunt vector. The PlantCARE database (http://bioinformatics.psb.ugent.be/webtools/plantcare/html/) was used for sequence analysis of the *VvPR1* promoter and revealed many motifs, sequences, and cis-elements responsible for gene regulation and expression in many eukaryotic promoters (Supplementary Figure S3; Supplementary Table S3). The cis-elements related to defense, hormones, and stress were as found in other plant promoters. The *VvPR1* promoter was enriched with CAAT-Box and TATA-box as follows: 1) light-responsive elements (AT1, LS7, AE-box, chs-CMA1a, Box4, and G-box), 2) stress-responsive elements (ARE, LTR, and MBS) respond to low temperature, drought, and anaerobic conditions 3) hormone-responsive elements (ERE, P-box, CGTCA-motif, and ABRE), 4) growth-associated elements (circadian and O2-site) that confer responsiveness to circadian control and zein metabolism regulation. Cis-acting elements (F-box and Unnamed-10) that had unclear functions were also found.

### GUS expression of *VvPR1* promoter against SA treatment

**FIGURE 7:**
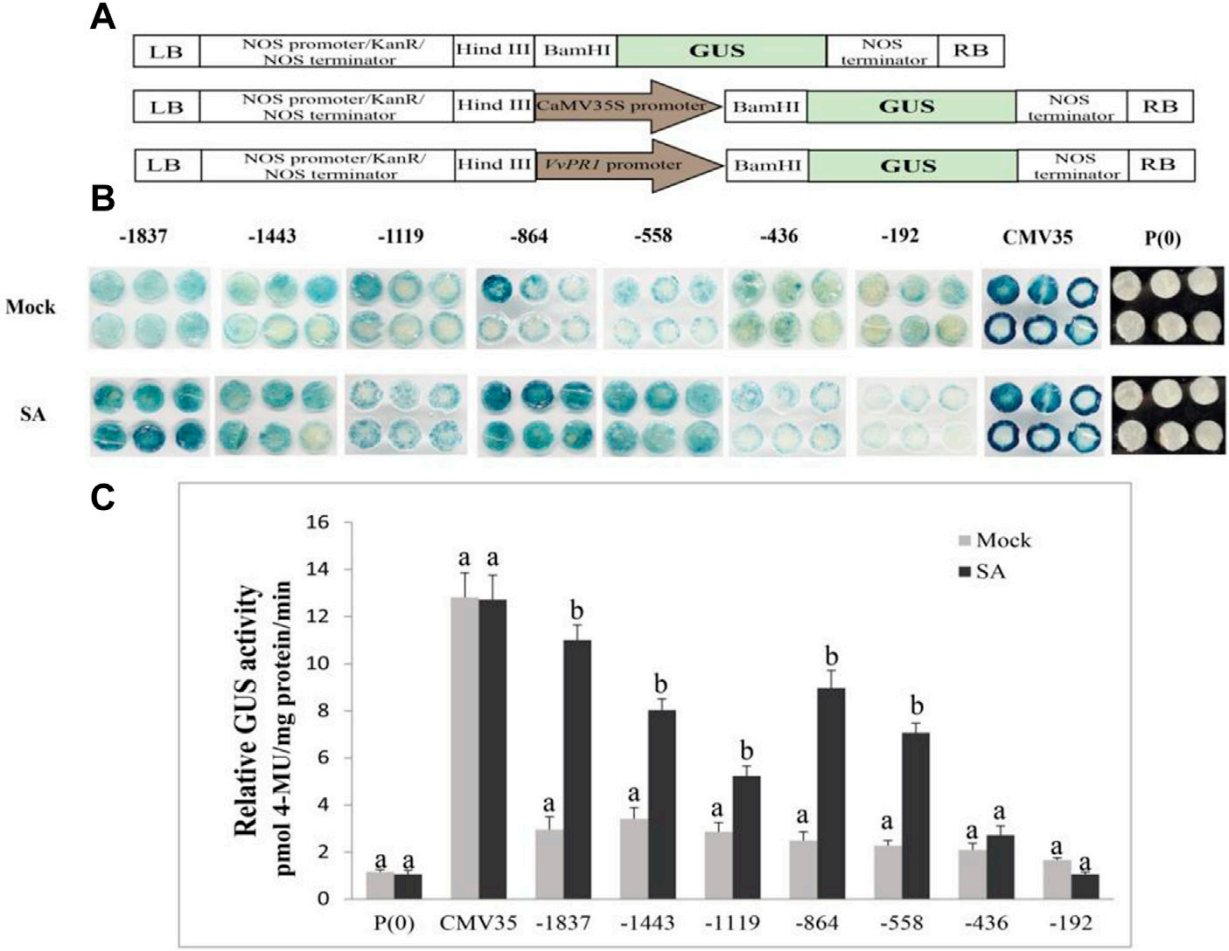
Schematic representation for the vector construction, histochemical staining, and fluorometric assay. **(A)** Promoter-GUS expression constructs showed the schematic structure. *P(0);* negative control; *P(35S);* positive control, and *VvPR1* promoter in the forward orientation, respectively. **(B)** Histochemical staining analysis; GUS activity in transiently transformed *N. benthamiana* leaves with serially deleted *VvPR1* promoter fragments (−1837, −1,443, −1,119, −845, −558, −436, and −192) bp against SA. **(C)** Fluorometric assay of *VvPR1* promoter fragments (−1837, −1,443, −1,119, −845, −558, −436, and −192) bp in response to SA in tobacco leaves through transient expression. Different letters on the bars showed a significant difference between SA-treated fragments and the control according to the least significant difference (LSD) test (*p* < 0.05).

The serially deleted fragments of the *VvPR1* promoter (−1,837 bp, −1,443 bp, −1,119 bp, −864 bp, −558 bp, −436 bp, and −192 bp) were cloned into the binary expression vector *pBI121::GUS* (Figure 7A). GUS expression was measured through histochemical staining and fluorometric assays in tobacco (*N. benthamiana*) plants. The above-mentioned constructs were infiltrated into the tobacco leaves and examined after 24 h of SA application by histochemical staining (Figure 7B) and fluorometric assays (Figure 7C). All deletion fragments of the promoter showed significantly higher GUS activity except −436 bp and −192 bp. The full-length promoter (−1,837 bp) exhibited the highest GUS protein activity, followed by the −864 bp, and −558 bp, which had shown significantly higher GUS activity with respect to the control. The −558 bp promoter fragment was found to be the shortest promoter fragment for the expression of the GUS reporter gene under SA treatment.

### GUS expression of *VvPR1* promoter against ABA treatment

**FIGURE 8:**
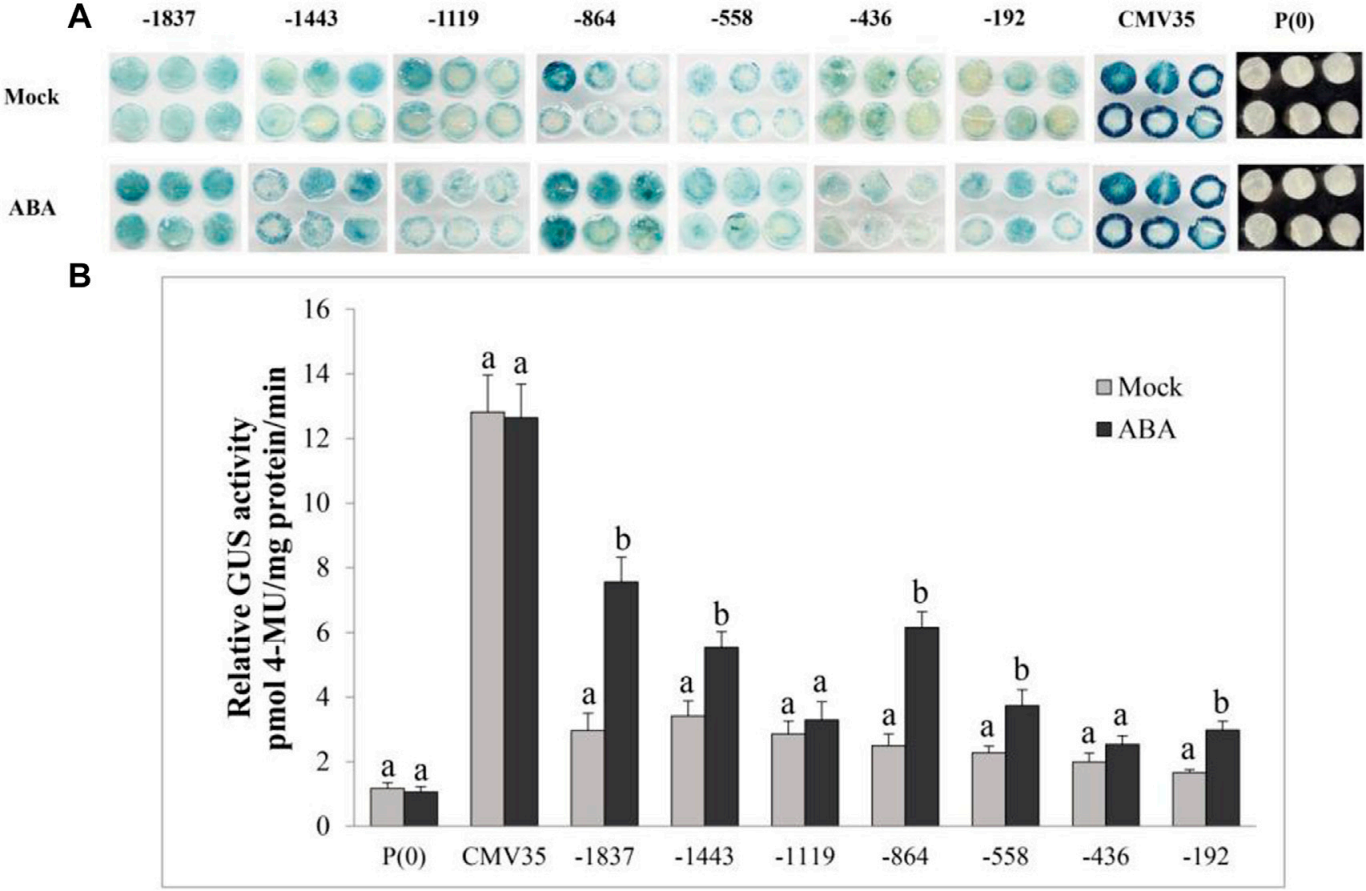
Histochemical staining and fluorometric assay of *VvPR1* in response to ABA. **(A)** Histochemical staining analysis; GUS activity in transiently transformed *N. benthamiana* leaves with serially deleted *VvPR1* promoter fragments (−1,837, −1,443, −1,119, −845, −558, −436, and −192) bp against ABA. **(B)** Fluorometric assay of *VvPR1* promoter fragments (−1,837, −1,443, −1,119, −845, −558, −436, and −192) bp in response to SA in tobacco leaves through transient expression. Different letters on the bars showed a significant difference between the SA-treated promoter fragments and the control according to the least significant difference (LSD) test (*p* < 0.05).

The effect of another plant hormone, ABA, on the activation of the *VvPR1* promoter was also investigated through a GUS assay in tobacco leaves harboring promoter-GUS chimeric constructs. The GUS expression of *VvPR1* promoter fragments from −1837 bp to −192 bp had induced significantly higher GUS activity with respect to the control except for −1,119 bp and −436 bp (Figures 8A,B).

## Discussion

Grape white rot disease is the major threat to *Vitis* species and grapevine cultivation in China. To identify the resistant germplasm for breeding and research purposes, pathogenesis tests were performed on the available germplasm. It was found that Chinese wild grape species *V. davidii* was resistant to the grape white rot disease (Figure 2) as was previously reported in our laboratory (Zhang et al., 2013; Zhang et al., 2019). Phytohormones such as SA have a key role in plants to respond to different environmental stresses and pathogen attacks (Alazem and Lin, 2015). The best defense-related hormone is known as SA (Ryals et al., 1994; Durrant and Dong, 2004a; Fu and Dong, 2013). When a pathogen attacks a plant, it induces the SA accumulation and the defense response (Vernooij et al., 1994; Sharma and Davis, 1997; Tsuda et al., 2008). In our current study, the amount of SA production in the resistant cultivar (*V. davidii*) was higher than in the susceptible (*V. vinifera*). We performed the transcriptome analysis on the susceptible grapevine cultivar treated with exogenous SA, hypothesizing that SA may induce the defense genes in grapevine plants. The RNA-seq analysis was conducted on SA-treated grapevine leaves and 511 DEGs were identified in which 12, 33, and 44 genes were down-regulated, and 15, 183, and 240 genes were upregulated after 12, 24, and 48 h of SA treatment, respectively. A higher number of DEGs were enriched in the GO terms and domains after 48 of SA treatment. Three domains from three GO terms, including cellular process, cellular anatomic entity, and catalytic activity, enriched a maximum number of downregulated and upregulated genes after 48 h of SA treatment on grapevine leaves. The top 20 KEGG pathways comprised most of the defense and immune related pathways; among them, phenylalanine metabolism, MAPK signaling pathway, Ras signaling pathway, Toll-like receptor signaling pathway, and alanine aspartate and glutamate metabolism that have a crucial role in plant disease resistance.

The SA level is elevated during the MTI and PTI response of the plant (Iwai et al., 2007; Garcion et al., 2008; Palmer et al., 2017). SA plays a vital role in the plant’s defense against biotrophic and semi-biotrophic pathogens (Fu and Dong, 2013). Moreover, exogenous SA treatment and its active analogs also induce defense mechanisms in plants against semibiotrophic and biotrophic pathogens (Lu, 2009). In plant defense, SA helps to encode the proteins related to antimicrobial activities through the induction of PR genes. To date, 17 PR families have been identified; among them, PR1, PR2, and PR5 are activated by biotrophic and semibiotrophic pathogens (Stintzi et al., 1993; Hoffmann-Sommergruber, 2000). SA also control the expression of PR1, PR2, and PR5 (Leah et al., 1991; Zhang et al., 2010); they are also used for the SA pathway as a marker. In the current study, PR1 and PR2 genes were upregulated after the SA treatment on the grapevine leaves at all time points. NPR1 is detected through the *Arabidopsis* mutants with an abolished expression of the PR gene (Cao et al., 1997). NPR1 is known as a master regulator of plant defense through the SA; it controls almost 98% of SA-mediated genes (Wang et al., 2006). In this study, NPR1 was also significantly upregulated from the leaf samples after the SA treatment. SA controls the translocation of NPR1 through the specific redox reactions (Mou et al., 2003). The oligomers of NPR1 formed by the intermolecular disulfide bond are found in the cytoplasm in the absence of infection or SA treatment, but after the SA treatment or infection, intermolecular bonds break, and monomers of NPR1 translocate into the nucleus, where they induce the expression of defense-related genes.

During the plant defense response, NPR1 regulated the expression of PR genes through cofactors known as TGAs because DNA-binding domains are missing on NPR1 (Zhang et al., 1999; Kesarwani et al., 2007). In *Arabidopsis,* it has been found that TGA2, 3, 5, 6, and 7 show interaction with NPR1 and NPR1 helps to bind TGA transcription factors on the as-1 element in the PR1 promoter region to induce the expression (Després et al., 2000; Després et al., 2003; Johnson et al., 2003). Additionally, *VvTGA2* TF was identified from the DEGs after the SA treatment on the grapevine leaves that may bind the promoter of *VvPR1* gene with *VvNPR1* to activate the plant defense response. NPR3 and NPR4 also bind SA but have been identified as negative regulators of plant defense, in contrast to NPR1, which plays a vital role in SA signaling (Zhang et al., 2006; Fu et al., 2012). NPR1 also works as an SA receptor through SA binding (Wu et al., 2012; Ding et al., 2018). During the plant defense, the NPR1 paralogues, NPR3 and NPR4, are SA receptors that bind SA with different affinities and function as adaptors of the Cullin 3 ubiquitin E3 ligase to mediate NPR1 degradation in an SA-regulated manner (Fu et al., 2012). Meanwhile, Ding et al. claimed that SA-based plant immunity was also accomplished independently by NPR3 and NPR4 (Fu et al., 2012; Ding et al., 2018).

After the RT-PCR of the random DEGs from the RNA-seq data, the *VvPR1* gene was selected for further study because PR1 is the SA marker gene for plant defense response. The promoter of the *VvPR1* gene was isolated from grapevine, and it was found that the *VvPR1* promoter was enriched with CAAT-boxes and TATA-boxes. Other cis-elements, such as stress, hormone, light, growth and development, and associated elements, were also detected from the *VvPR1* promoter. In this study, the *VvPR1* promoter was enriched with TATA boxes, especially up to −800 bs upstream from the ATG. TATA-boxes are abundant in stress-related genes and are absent in essential genes (Tirosh et al., 2006; Walther et al., 2007). They are important with a variable and rapid gene induction (Newman et al., 2006; Roelofs et al., 2010; Vos et al., 2015). A previous study of *Arabidopsis* found that the PR-1 promoter contains cis-acting regulatory elements responsible for its induction on exogenous 2,6-dichloroisonicotinic acid (INA) and SA (Lebel et al., 1998). Hormones, different stresses, and pathogens activate the pepper PR1 promoter, possibly by transactivating the CARAV1 and CAZFP1 transcription factors (Hong et al., 2005).

Phytohormones (SA, JA, and ABA) are well-known major signaling components in plant defense signaling networks (Dong, 1998). SA is an important biomolecule in disease resistance that recognizes the pathogen effectors directly or indirectly and induces local resistance and systematic resistance against the biotrophic pathogens (Durrant and Dong, 2004b). In this study, the *VvPR1* promoter fragments showed high GUS induction from −1,837 bp to −558 bp against SA. The expression of GUS of the −1,443 bp of *VvPR1* promoter increased 2.35-fold with respect to the control under the treatment of SA. In addition, the −1,119, −864, and −558 bp deletion fragments resulted in 1.85-, 3.60-, and 3.11-fold increases in GUS expression by the SA treatment, respectively. This indicated that the minimal cis-regulatory elements important for the molecular response to SA might be present in the 1282-bp region between −1837 and −558 of the *VvPR1* promoter. The expression of the *VvPR1* promoter upon the SA treatment may be due to the presence of the TCA-element, *activation sequence-1* (as-1), and the LS7 cis-acting elements. In this study, the *VvPR1* promoter contained the TCA-element, as-1 and LS7 cis-acting elements at -1,472 bp, −1,428 bp, and −520 bp, respectively. Previous studies provide strong evidence that the TCA element is involved in SA signaling and PR-1 promoter induction by providing the site for TGA transcription factors and recruitment of NPR1 (Pastuglia et al., 1997). On the accumulation of SA in the cell, NPR1 monomers translocate in the nucleus (Zhang et al., 1999; Kinkema et al., 2000; Zhou et al., 2000), where they interact with TGA transcription factors and activate the expression of the PR-1 gene and subsequently SAR activation (Zhang et al., 2003; Kesarwani et al., 2007). Activation sequence-1 (as-1) is an SA-inducible cis-element in promoters of some pathogenesis-related genes in *Arabidopsis thaliana* PR-1 and tobacco PR-1a. In the promoter of PR-1a, tobacco possesses an as-1-like element with inverted TGACG motifs, a binding site for TGA transcription factors (Strompen et al., 1998; Niggeweg et al., 2000; Grüner et al., 2003). Further studies have shown that LS7 is also involved in SA-inducible PR-1 gene expression (Pape et al., 2010).

ABA is an important plant hormone that responds to biotic and abiotic stresses (Ton et al., 2009; Sah et al., 2016; Alazem et al., 2017). On the application of ABA, several transcription factors bind to target genes and respond to the stress (Song et al., 2016). In this study, the GUS protein expression has been induced upon ABA treatment from all deleted fragments of *VvPR1* except −1,119 and −436; this indicated that these regions might contain some ABA-repressing elements. The induction of GUS with ABA treatment may be due to the presence of ABRE (a cis-acting element involved in the abscisic acid responsiveness) (Shinozaki and Yamaguchi-Shinozaki, 1997) that is located at −163 bp upstream from the transcription initiation start site. It was also found that ABA promotes proteasome-mediated degradation of the transcription coactivator NPR1 in *Arabidopsis thaliana* (Ding et al., 2016) that may express the PR-1 gene under ABA treatment. Overall, the GUS protein expression by the *VvPR1* promoter under the treatment of ABA was found to be low compared to SA; this low activation may be due to the presence of the low number of ABRE cis-acting elements in the *VvPR1* promoter.

## Conclusion

**FIGURE 9:**
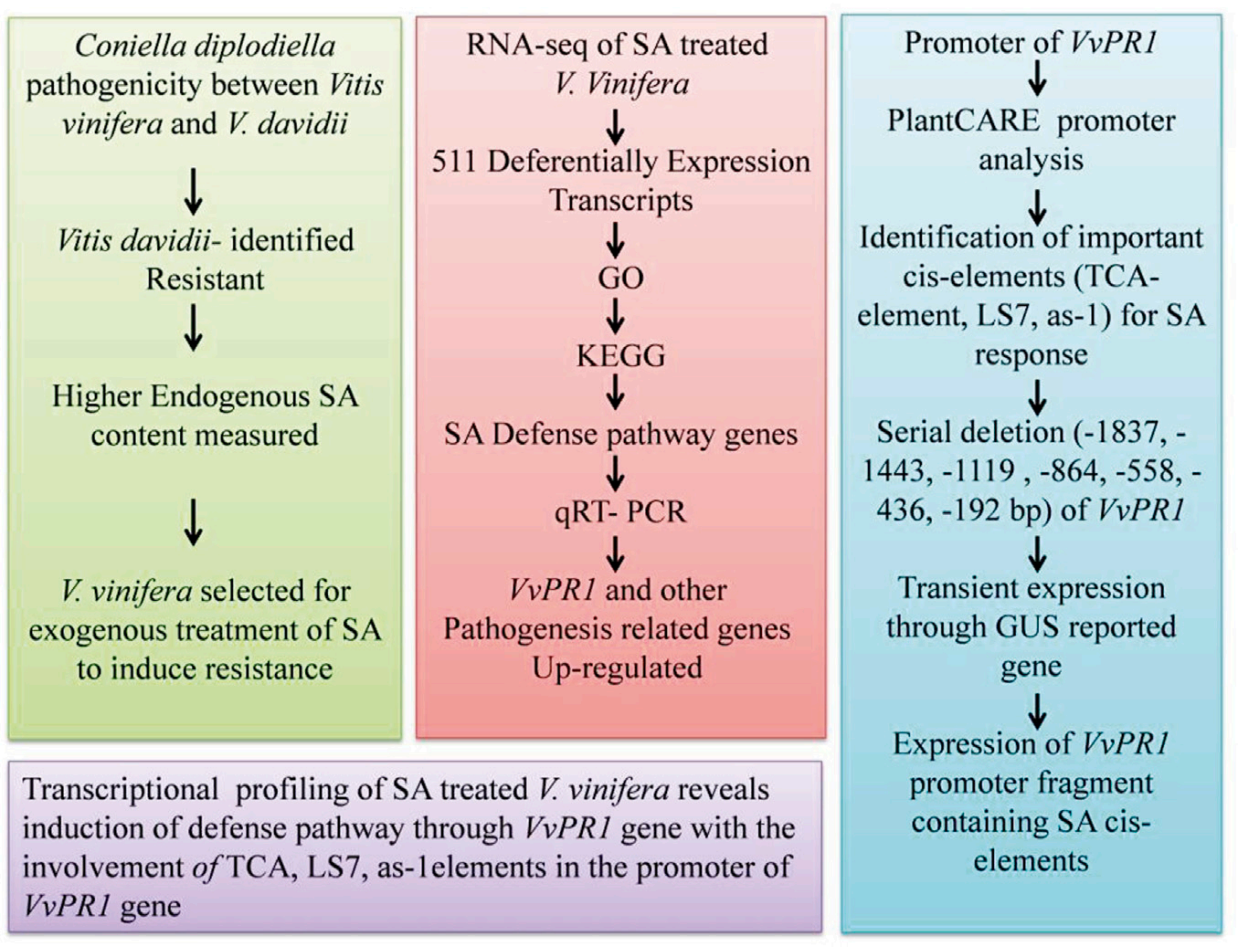
Flow chart of study pattern showing the different steps of inducing the pathogenicity of grape white rot, the RNA-seq data analysis, promoter analysis through serial deletion of promoter, and the conclusion.

In conclusion, SA levels were found to be higher in resistant grapevine (*V. davidii*) than in susceptible grapevine (*V. vinifera*) after a pathogenicity test against white rot disease. After the transcriptome analysis, the GO and KEGG pathway analysis revealed that DEGs include important genes related to the SA defense pathway known as *VvPR1*. The promoter of the *VvPR1* gene was also analyzed through serial deletion of the *VvPR1* promoter with the GUS reporter gene. Deletion analysis showed that the region between −1837 bp and −558 bp of the *VvPR1* promoter expressed significantly high GUS protein with respect to the control. On the basis of these results, this region was deduced to be an important part of the *VvPR1* promoter, which contained the most important cis-elements (TCA-elements, LS7, and *as-1*) responsible for *VvPR1* gene expression in response to SA application (Figure 9). Overall, the current study validated the available information about SA-mediated defense responses in grapevine species that are susceptible to various diseases.

## Data Availability

The sequencing data has been deposited into the NationalGenomics Data Center (Accession Number: PRJNA845078; link: https://www.ncbi.nlm.nih.gov/search/all/?term=PRJNA845078.
