# Supplementary material for: Transcriptome analysis reveals pathogenesis-related gene 1 pathway against salicylic acid treatment in grapevine (Vitis vinifera L)
Source: Front Genet. 2022 Oct 20;13:1033288. doi: 10.3389/fgene.2022.1033288 (PMC9631220; doi:10.3389/fgene.2022.1033288)
Supplement: Supplementary file 1 [file DataSheet1.docx]

Supplementary Material

.

## Supplementary Figures
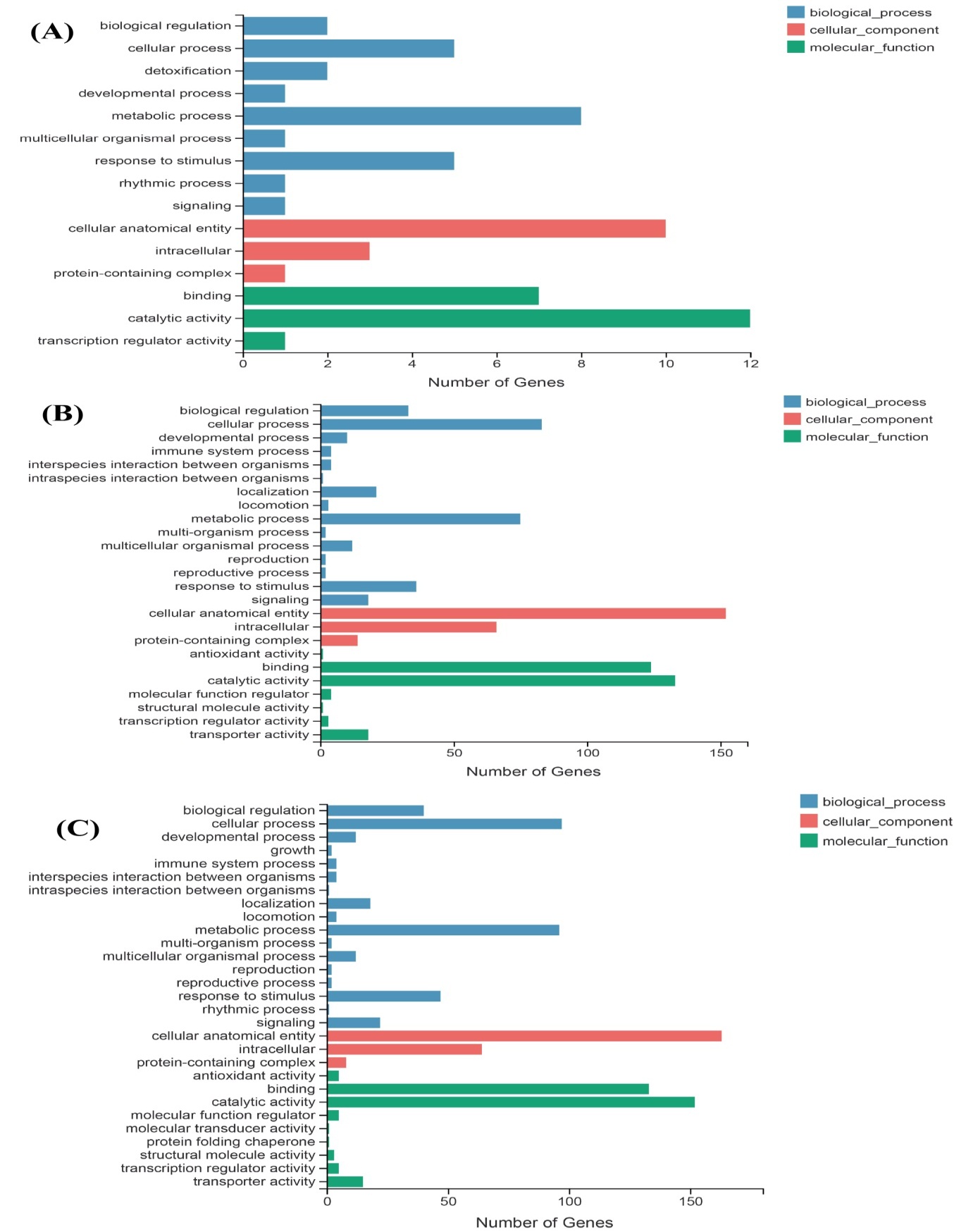


**Supplementary Figure 1.** Gene Ontology analysis of RNA-seq data. **(A)** GO analysis after 12 h of SA treatment. **(B)** GO analysis after 24 h of SA treatment. **(C)** GO analysis after 48 h of SA treatment.


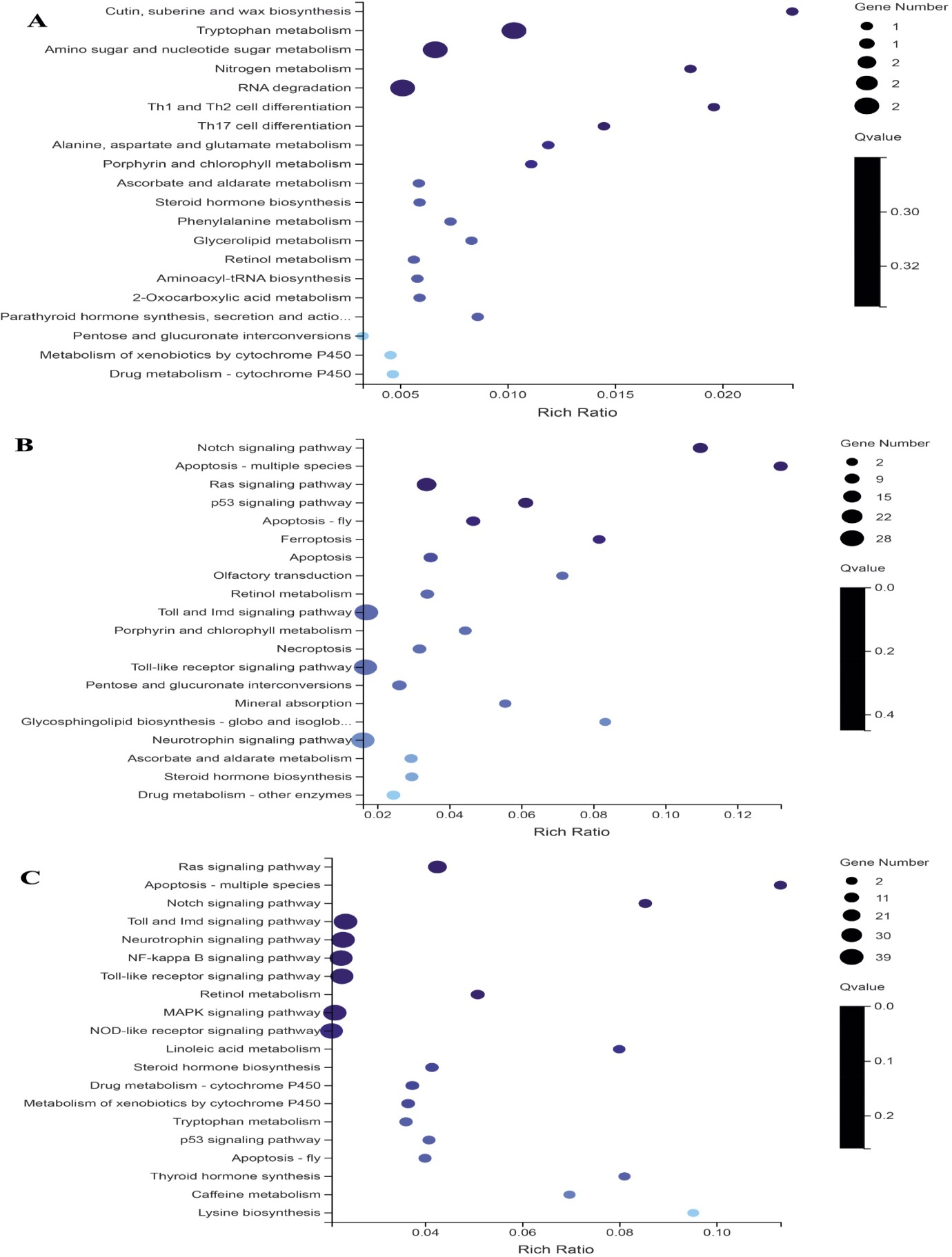
 **Supplementary Figure 2.** Kyoto Encyclopedia of Genes and Genomes (KEGG) analysis of DEGs at all-time points of SA treatment through enrichment analysis: (**A**) 12 h of SA treatment, (**B**) 24 h SA treatment, and (**C**) 48 h of SA treatment.


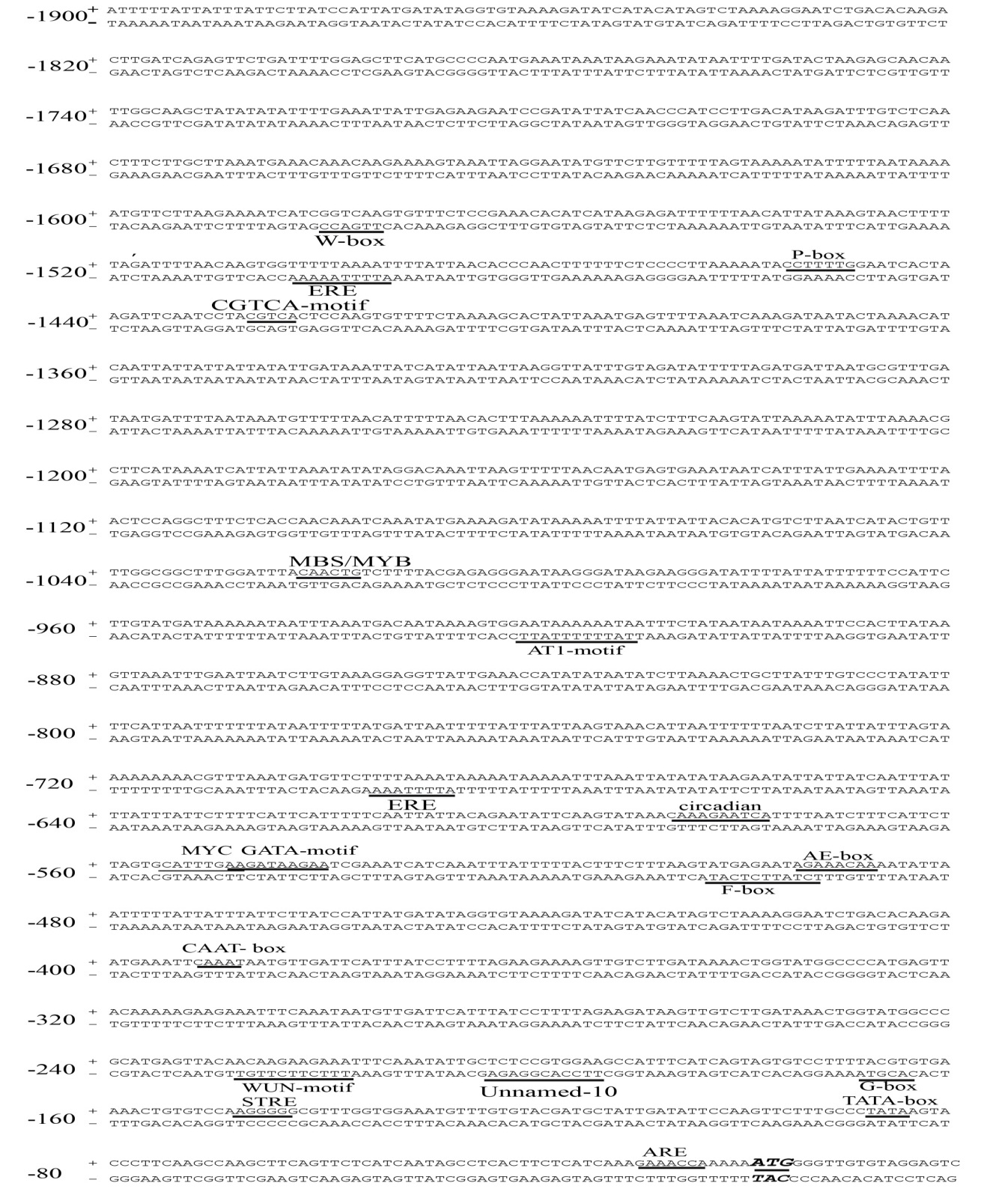


**Supplementary Figure 3.** The 5’ upstream nucleotide sequence starting from the transcription start site of the *VvPR1* gene containing the putative promoter and predicted cis-acting elements. The Underline sequence showed the sequence of putative cis-regulatory elements of the promoter. Promoter contained **(a)** hormone-responsive elements (ERE, P-box, and CGTCA-motif) **(b)** light-responsive elements (AE-box,AT1,G-box and chs-CMA1a ) **(c)** stress-responsive elements (MBS, MYC, and ARE) **(d)** and other growth associated elements (O2-site, circadian) that involved in zein metabolism regulation and circadian control. F-box and Unnamed_10 were found cis-elements with unclear function

1. **Supplementary Tables**
2. **Supplementry** Table S1 Primers list used for qRT-PCR.

| **Sr #** | **Name of Primers** | **Primers** | **(Forward/Reverse)** |
| --- | --- | --- | --- |
| 1 | ***VvPR1*** | ATGGGGTTAGCCCTAGCTCA | F |
|  |  | CGCCAATCCTCTGGTTAGCA | R |
| 2 | ***VvWRKY2*** | GGTCTCCGCTTCTTCCAAGG | F |
|  |  | AGAAACCTGGGGAATCGAGC | R |
| 3 | ***VvEDS1*** | GAAATCGTGAACAGGGCTGC | F |
|  |  | AAGGTGGGTGCGTAAACCAA | R |
| 4 | ***VvNPR1*** | CGGAGAATCTCGAGTCGGTG | F |
|  |  | ACACGGCCTTGAAAAACACG | R |
| 5 | ***VvBAK1*** | GCTGAAGGTGATTGGGACGA | F |
|  |  | CAGGAGGCATTGTGGTGGAA | R |
| 6 | ***VvSTB4.14*** | TGCTGCAAGCACCATGGATA | F |
|  |  | ACTGGTGCAGTACTTGGCTC | R |
| 7 | ***VvPR2*** | CATTGGTAACACCCGGGACA | F |
|  |  | AACAACCTGCAAAGAACCGC | R |
| 8 | ***VvCHI4D*** | GCAGTGGTAGCTCGGTTTCT | F |
|  |  | TCGCGCTTATTAGCATCGGT | R |
| 9 | ***VvTGA2*** | TGCCGAGCTTCATTCCTCAG | F |
|  |  | GTTGGGTCAGCTTCATCCGA | R |
| 10 | ***VvACT1*** | CCACCTTGATCTTCATGCTGC | F |
|  |  | CACCTCCCCATGCTATCCTTCGTC | R |

**Supplementry Table 2.** List of primers used for cloning and serial deletion of promoter

**Name of Primers Primers (Forward/Reverse)**

*VdPR1* 5’TGATAGTCTAGTAGCTGTGAATTTATCCTTGATCAG 3’ F

5’ TGGAGAGTTCTGGGCACAGC 3’ R

[-1837 to -20] 5’ATGACCATGATTACGCCAAGCTTACCCATCCTTGACATAAGATTTGTCTCAAC 3’F

5’GACTGACCACCCGGGGATCCTTGATGAGAAGTGAGGCTATTGATGAGAAC 3’R

-1837 5’ACCATGATTACGCCAAAGCTTGATCAGAGTTCTGATTTTGGAGCTTCATGC 3’F

-1443 5’ TATGACCATGATTACGCCAAGCTTCTAAGATTCAATCCTACGTCACTCC 3’ F

-1119 5’ TATGACCATGATTACGCCAAGCTTACTCCAGGCTTTCTCACCAA 3’ F

-864 5’ TATGACCATGATTACGCCAAGCTTTCTTGTAAAGGAGGTTATTGAAACCATA 3’ F

-558 5’TATGACCATGATTACGCCAAGCTTGTGCATTTGAAGATAAGAATCGAAATCA 3’ F

-436 5’TATGACCATGATTACGCCAAGCTTGATATCATACATAGTCTAAAAGGAATCTGAC 3’F

-192 5’ TATGACCATGATTACGCCAAGCTTGCCATTTCATCAGTAGTGTCC 3’ F

R^a^ 5’ AAGGGACTGACCACCCGGGGATCCGACTCCTACACAACCCCAT 3’ R

R^a^ is the reverse primer sequence for the deletion fragments except for the (-1837 to -20) primer. Underline below the nucleotide sequence showed the restriction site for restriction enzymes.

**Supplementary Table 3** Predicted cis-elements in *VvPR1* promoter sequence by PlantCARE and PLACE promoter databases

| **Motif** | **Copies** | **Sequence** | **Function** |
| --- | --- | --- | --- |
| TATA-box | 42 | TATA | Core promoter element |
|  |  | TATATA |  |
|  |  | ATATAT |  |
| ABRE | 1 | ACGTG | Abscisic acid responsiveness Cis- element |
| CGTCA-motif | 2 | CGTCA | MeJA-responsiveness Cis- element |
| ERE | 2 | ATTTTAAA | Ethylene responsive |
| G-box | 1 | TACGTG | Cis-acting regulatory element involved in light responsiveness |
| LS7 | 1 | CAGATTTATTTTTA | Part of a light responsive element |
| MBS | 2 | CAACTG | MYB binding site involved in drought-inducibility |
| ARE | 3 | AAACCA | Cis- element essential for the anaerobic induction |
| AT1-motif | 2 | AATTATTTTTTATT | Part of a light responsive module |
| CAAT-box | 29 | CAAT | Common cis- element in promoter and enhancer regions |
|  |  | CCAAT |  |
|  |  | CAAAT |  |
| GATA-motif |  | AAGATAAGATT | Cis- element required for high level light regulated and tissue specific |
| TCA-element | 1 | CCATCTTTTT | Cis-acting element involved in salicylic acid responsiveness |
| W box | 1 | TTGACC | Defense related ( recognized especially by Salicylic acid (SA)-induced WRKY DNA binding protein) |
| Circadian | 1 | CAAAGATATC | Cis- element involved in circadian control |
| as-1 | 1 | TGACG | Binding site for TGA transcription factors |
| LTR | 1 | CCGAAA | Cis- involved in low-temperature responsiveness |
| MYC | 3 | CAATTG | MYC recognition elements |
| STRE | 2 | AGGGG | Stress responsive element |
| Circadian | 1 | CAAAGATATC | Cis- element involved in circadian control |
